# Pre-Activation of Mitophagy Protects Against Hyperbaric Oxygen-Induced Central Nervous System Oxygen Toxicity

**DOI:** 10.3390/ijms27114982

**Published:** 2026-05-30

**Authors:** Wei Ding, Qi Zhu, Houyu Zhao, Guanning Wei, Guoyang Huang, Jing Shi, Longfei Wang, Nan Zhao, Jin Ding, Yiqun Fang

**Affiliations:** 1 Naval Medical Center, Naval Medical University, Shanghai 200433, China; 13255589299@163.com (W.D.); imzhuqi@163.com (Q.Z.); zhaohouyuecho@163.com (H.Z.); huangswa@163.com (G.H.); fdshijing_sj@126.com (J.S.); wanglongfeittxn@163.com (L.W.); zhaonan350@163.com (N.Z.); 2 National Key Laboratory of Immunity and Inflammation, Shanghai 200433, China; 3 Translational Medical Research Center, Naval Medical University, Shanghai 200433, China; weiguanning@hotmail.com

**Keywords:** hyperbaric oxygen, central nervous system oxygen toxicity (CNS-OT), mitophagy, neuroprotection

## Abstract

Central nervous system oxygen toxicity (CNS-OT) is a major complication of hyperbaric oxygen (HBO) characterized by seizures and neuronal damage, yet the underlying mechanisms remain incompletely understood. Using male Sprague Dawley rats (n = 6 per group) and HT22 neurons exposed to either HBO (6 ATA, 100% O_2_) or hyperbaric normoxia (HNO), our results demonstrate that HBO, but not HNO, caused mitochondrial structural damage and loss of mitochondrial membrane potential (ΔΨm). Transcriptomic analysis revealed enrichment of apoptosis and mitogen-activated protein kinase (MAPK) signaling pathways. Using HeLa cells stably overexpressing Parkin and Mito-Keima, a pH-sensitive mitochondrial probe system for monitoring mitophagy, we observed that mitophagic flux was initiated but proceeded too slowly to clear damaged mitochondria in a timely manner in HBO-exposed neurons. Pharmacological preconditioning to activate mitophagy enabled the prompt elimination of dysfunctional mitochondria and rescued HBO-induced mitochondrial dysfunction and cell death. In vivo, everolimus treatment promoted timely mitophagic clearance, prolonged seizure latency, attenuated ΔΨm loss, and suppressed p-p38 activation. These findings demonstrate that HBO exposure disrupts mitochondrial homeostasis and activates pro-apoptotic MAPK signaling. Meanwhile, endogenous mitophagy is initiated but fails to clear damaged mitochondria in a timely manner. Pre-activation of mitophagy by everolimus enables the timely clearance of damaged mitochondria, protecting against CNS-OT and highlighting a promising therapeutic strategy.

## 1. Introduction

Hyperbaric oxygen (HBO) technology plays an indispensable role in ensuring the safety of personnel operating in extreme environments such as deep-sea diving and high-altitude stationing, as well as in the treatment of various medical conditions, including decompression sickness, carbon monoxide poisoning, and combat-related injuries [1,2]. However, prolonged exposure to HBO can precipitate oxygen toxicity, defined as a pathological condition caused by exposure to partial pressures of oxygen exceeding physiological tolerance. Among its manifestations, central nervous system oxygen toxicity (CNS-OT) is particularly critical, presenting with symptoms ranging from visual disturbances, nausea, muscle twitching, and vertigo to rapidly progressing generalized tonic-clonic seizures, coma, and even death [3,4,5]. In the present study, CNS-OT was identified by the occurrence of generalized tonic–clonic seizures, and seizure latency was recorded as the primary behavioral endpoint. This sudden and unpredictable neurological event severely limits the oxygen partial pressure and exposure duration that can be safely employed in hyperbaric oxygen therapy, while also posing a significant threat to the lives of individuals engaged in deep-sea diving, hyperbaric operations, and other specialized occupations. Therefore, elucidating the core molecular mechanisms underlying CNS-OT and developing effective preventive strategies represent a major scientific and practical imperative to overcome current safety limitations, expand the clinical applications of HBO therapy, and protect the health of personnel operating in extreme environments.

While hyperbaric oxygen therapy has been extensively studied for its therapeutic applications, research specifically addressing the neurotoxic mechanisms of hyperbaric hyperoxia remains scarce [2,6]. The mechanisms underlying CNS-OT remain incompletely understood. Excessive reactive oxygen species (ROS) production is widely recognized as a central pathogenic event [7,8,9]; under hyperbaric hyperoxic conditions, the electron transport chain within mitochondria becomes overwhelmed, leading to elevated superoxide and other ROS generation. This oxidative stress triggers a cascade of cellular damage, including lipid peroxidation, protein oxidation, and DNA damage, ultimately contributing to neuronal dysfunction and cell death [8,10].

Mitophagy, the selective autophagic clearance of damaged or dysfunctional mitochondria, plays an essential role in maintaining mitochondrial homeostasis. Upon mitochondrial damage, the PTEN-induced putative kinase 1 (PINK1)/Parkin pathway is activated, targeting depolarized mitochondria for engulfment by autophagosomes and subsequent lysosomal degradation. This process prevents the accumulation of ROS-producing, dysfunctional mitochondria that would otherwise trigger apoptotic cell death. Accumulating evidence from various neurological disorders has demonstrated that activating or restoring proper mitophagic flux exerts significant neuroprotective effects [11,12,13]. For instance, upregulation of PINK1/Parkin- or FUN14 domain-containing 1 (FUNDC1)-mediated mitophagy has been shown to improve neurological outcomes in ischemic stroke models [14,15]; similarly, enhancing mitophagy helps clear damaged mitochondria and delays disease progression in Alzheimer’s and Parkinson’s diseases [16,17,18,19]. Everolimus, an mTOR inhibitor and autophagy activator capable of crossing the blood-brain barrier, was used to activate brain mitophagy, aiming to enhance the timely clearance of damaged mitochondria and protect against CNS-OT.

Despite these advances, the role of mitophagy in HBO-induced neurotoxicity has not been systematically investigated. Whether endogenous mitophagy is promptly engaged following HBO exposure, and whether pre-activation of mitophagy could confer protection against CNS-OT, remain unknown. In this study, we systematically investigated the temporal dynamics of mitophagy and its functional consequences in both in vivo and in vitro models of HBO exposure. We further examined whether pharmacological pre-activation of mitophagy with everolimus could mitigate mitochondrial dysfunction, suppress apoptotic signaling, and prolong seizure latency, thereby establishing mitophagy activation as a potential preventive strategy for CNS-OT.

## 2. Results

### 2.1. HBO Exposure Triggers Mitochondrial Injury and Autophagic Sequestration in Rat Hippocampus

**Figure 1:**
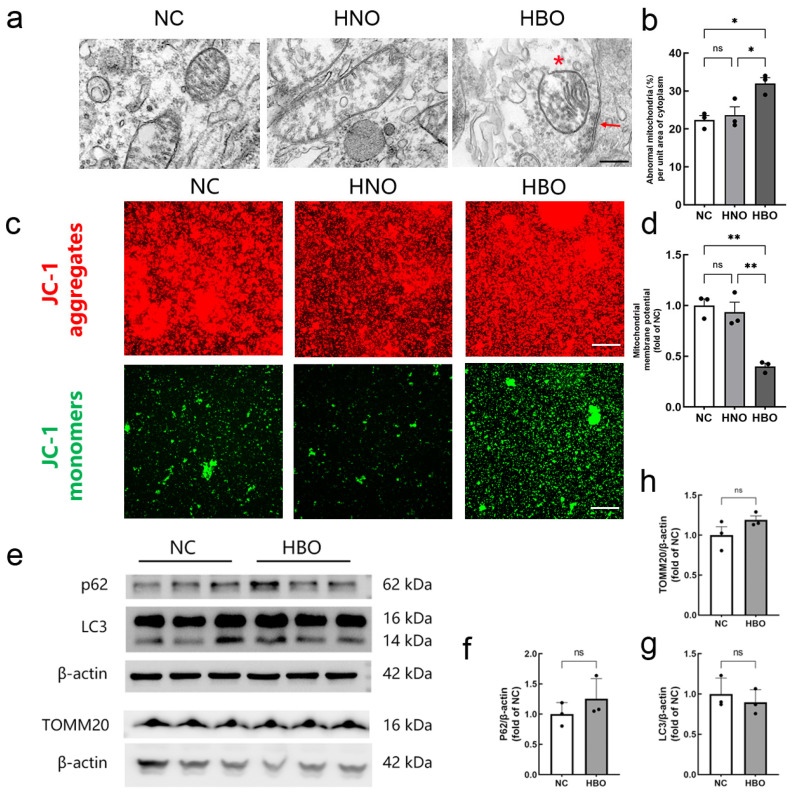
HBO exposure induces mitochondrial damage and autophagic sequestration in the rat hippocampus: (**a**,**b**) Representative transmission electron microscopy images of hippocampal neurons from the NC, HNO, and HBO groups. The red asterisk (*) indicates a double-membrane discontinuity. The red arrow points to an autophagosome-like double-membrane structure surrounding damaged mitochondria. (**c**,**d**) Assessment of mitochondrial membrane potential (ΔΨm) using JC-1 staining. Representative JC-1 fluorescence images (**c**) and quantitative analysis of the red/green fluorescence ratio (**d**) show a significant reduction in the HBO group compared to the NC and HNO groups. Scale bar, 400 μm. (**e**–**h**) Western blot analysis of mitophagy-related proteins in hippocampal tissues immediately after HBO exposure. Representative immunoblots (**e**) and quantitative analysis of p62 (**f**), LC3-II/LC3-I ratio (**g**), and TOMM20 (**h**) show no significant differences between the HBO and NC groups. Data are presented as mean ± SEM. * *p* < 0.05, ** *p* < 0.01 vs. indicated groups; n = 3 per group. NC, normobaric normoxia; HNO, hyperbaric normoxia; HBO, hyperbaric oxygen; ns: not significant.

Transmission electron microscopy revealed normal mitochondrial morphology in hippocampal neurons from the NC (normobaric normoxia) group, with intact cristae and uniform matrix density. Notably, to distinguish the effects of hyperbaric pressure itself from those of hyperoxia, we included a hyperbaric normoxia (HNO, 0.6 MPa nitrogen-oxygen mixture with normobaric oxygen partial pressure) control group. The HNO group also exhibited normal mitochondrial ultrastructure. In contrast, rats exposed to HBO (hyperbaric hyperoxia, 0.6 MPa 100% O_2_) exhibited marked mitochondrial abnormalities, including swelling, disorganization of cristae, and matrix vacuolization (Figure 1a,b). To assess the functional consequences of HBO-induced mitochondrial damage, we measured ΔΨm using JC-1 staining. The red/green fluorescence ratio was significantly reduced in hippocampal tissues from HBO-exposed rats compared to the NC and HNO groups (Figure 1c,d), indicating mitochondrial depolarization.

Notably, autophagosome-like double-membrane structures surrounding mitochondria were occasionally observed in the HBO group (Figure 1a, arrow), suggesting attempted sequestration of damaged mitochondria. Such structures were rarely detected in NC or HNO groups, indicating that HBO stress triggers mitochondrial damage and initiates autophagic responses. To further characterize the autophagic response, we examined the expression of mitophagy-related proteins by Western blotting. The microtubule-associated protein 1 light chain 3 (LC3)-II/LC3-I ratio, an indicator of autophagosome formation, showed no significant change in the HBO group compared to the NC group immediately after exposure (Figure 1e,g). Consistently, P62 levels remained unchanged (Figure 1e,f), suggesting that autophagic flux had not yet been completed at this early time point. Additionally, translocase of the outer mitochondrial membrane 20 (TOMM20), a mitochondrial outer membrane protein that reflects mitochondrial mass and is typically degraded during mitophagy, showed no significant reduction (Figure 1e,h), further indicating that clearance of damaged mitochondria had not yet occurred.

### 2.2. Transcriptomic Analysis Reveals Enrichment of Apoptosis and Mitogen-Activated Protein Kinase (MAPK) Signaling Pathways Following HBO Exposure

**Figure 2:**
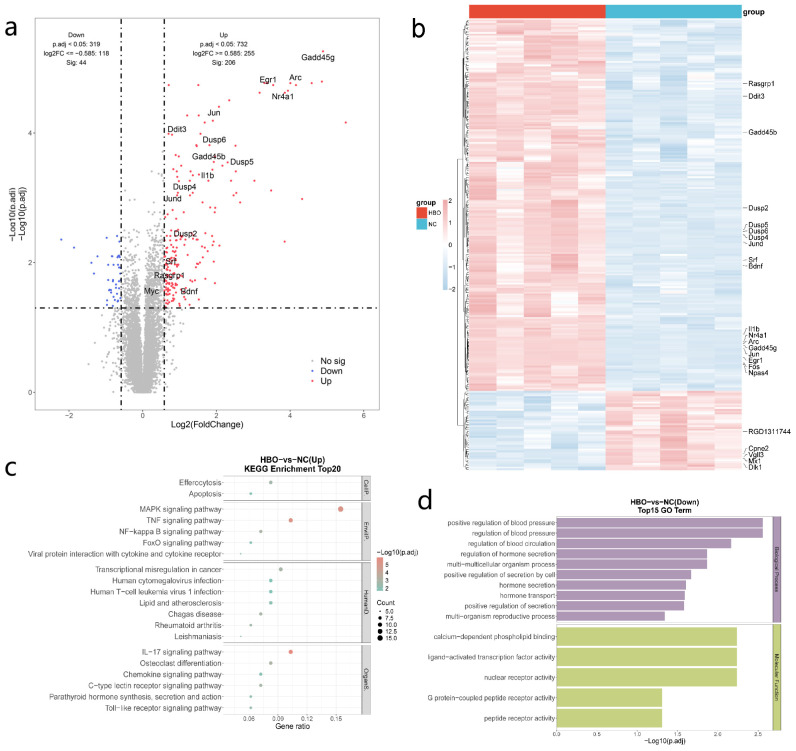
Transcriptomic analysis reveals enrichment of apoptosis and MAPK signaling pathways following HBO exposure: (**a**) Volcano plot showing differentially expressed genes between the NC and HBO groups. (**b**) Hierarchical clustering heatmap demonstrating distinct separation of gene expression profiles between the NC and HBO groups. (**c**) KEGG pathway enrichment analysis of differentially expressed genes, highlighting the MAPK signaling pathway, TNF signaling pathway, NF-κB signaling pathway, FoxO signaling pathway, and apoptosis pathway. (**d**) GO enrichment analysis of biological processes and molecular functions in downregulated genes. Data are derived from RNA sequencing of hippocampal tissues from the NC and HBO groups. NC, normobaric normoxia; HBO, hyperbaric oxygen.

To elucidate the molecular mechanisms underlying HBO-induced neurotoxicity, we performed RNA sequencing on hippocampal tissues from the NC and HBO groups. Differential expression analysis identified a substantial number of genes significantly altered following HBO exposure (Figure 2a). Hierarchical clustering analysis revealed distinct separation between the NC and HBO groups (Figure 2b).

The Kyoto Encyclopedia of Genes and Genomes (KEGG) pathway enrichment analysis of differentially expressed genes revealed significant enrichment of multiple signaling pathways (Figure 2c). Notably, the MAPK signaling pathway was among the most significantly enriched pathways, along with the tumor necrosis factor (TNF) signaling pathway, nuclear factor kappa B (NF-κB) signaling pathway, and forkhead box O (FoxO) signaling pathway. Additionally, pathways associated with inflammatory responses, including the interleukin-17 signaling pathway, chemokine signaling pathway, Toll-like receptor signaling pathway, and C-type lectin receptor signaling pathway, were also significantly enriched. Other enriched pathways included those related to infectious diseases, immune-related conditions, and metabolic or cardiovascular processes. The enrichment of the apoptosis pathway further supported the involvement of cell death programs in HBO-induced neurotoxicity.

Gene ontology (GO) enrichment analysis of biological processes revealed that the downregulated genes were predominantly associated with regulation of blood pressure, regulation of blood circulation, regulation of hormone secretion, hormone transport, and positive regulation of secretion (Figure 2d). Terms related to multicellular organism processes and multi-organism reproductive processes were also enriched, reflecting the systemic nature of the response to hyperbaric stress. GO molecular function analysis identified enrichment of calcium-dependent phospholipid binding, ligand-activated transcription factor activity, nuclear receptor activity, G protein-coupled peptide receptor activity, and peptide receptor activity (Figure 2d). These findings suggest that calcium signaling and transcriptional regulation play important roles in HBO-induced neuronal responses.

Collectively, these transcriptomic data demonstrate that HBO exposure triggers robust activation of MAPK, TNF, and NF-κB signaling pathways, upregulates pro-apoptotic gene expression, and induces a transcriptional program associated with inflammation and cellular stress, providing a molecular basis for further investigation into the mechanisms underlying HBO-induced neurotoxicity.

### 2.3. HBO Exposure Induces Mitochondrial Dysfunction and Apoptosis in HT22 Hippocampal Neurons

Based on the in vivo observations of mitochondrial damage and autophagic membrane recruitment, along with transcriptomic enrichment of apoptosis and MAPK pathways, we next examined the direct cellular effects of HBO using HT22 hippocampal neurons. Cells were exposed to NC, HNO (0.6 MPa nitrogen–oxygen mixture with normobaric oxygen partial pressure), or HBO (0.6 MPa 100% O_2_) for 1 h, and mitochondrial function was assessed at the indicated time points after decompression.

**Figure 3:**
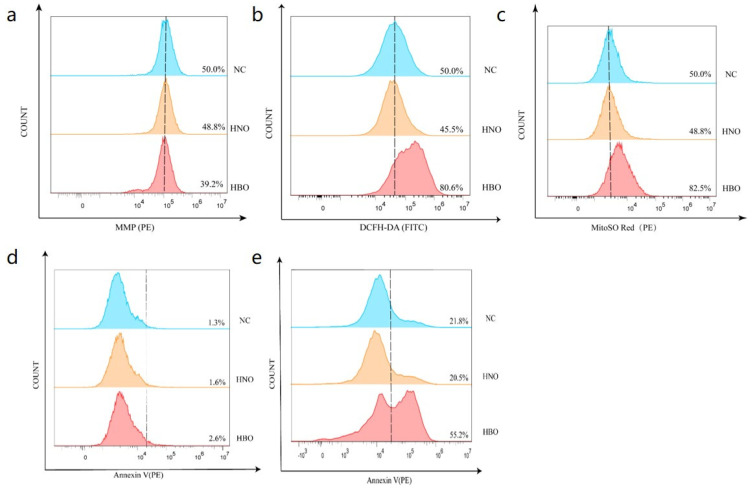
HBO exposure induces mitochondrial dysfunction and apoptosis in HT22 hippocampal neurons: (**a**) Flow cytometry analysis of mitochondrial membrane potential (ΔΨm) measured by TMRE staining. (**b**) Flow cytometry analysis of total ROS measured by DCFH-DA staining. (**c**) Flow cytometry analysis of mitochondrial ROS measured by MitoSOX Red staining. (**d**,**e**) Annexin V-PE staining for apoptosis at 0 h and 6 h post-exposure. NC, normobaric normoxia; HNO, hyperbaric normoxia; HBO, hyperbaric oxygen; MMP, mitochondrial membrane potential.

Mitochondrial membrane potential (ΔΨm) was evaluated using TMRE staining, a membrane potential-driven cationic probe that accumulates in healthy mitochondria. Flow cytometry analysis revealed that HBO exposure caused a significant decrease in the percentage of TMRE-positive cells, from 50% in the NC group to 39.2% in the HBO group, indicating marked mitochondrial depolarization (Figure 3a). In contrast, the HNO group showed no significant change in ΔΨm compared to NC controls, confirming that the observed mitochondrial dysfunction is specifically attributable to hyperbaric hyperoxia rather than pressure alone. We also assessed total and mitochondrial ROS levels immediately after decompression and removal from the chamber using DCFH-DA and MitoSOX Red staining in living cells, respectively. Flow cytometry analysis revealed a significant increase in both DCFH-DA-positive cells and MitoSOX Red-positive cells in the HBO group compared to NC controls (Figure 3b,c), indicating elevated total ROS production with mitochondria as a major source. No significant changes in ROS levels were observed in the HNO group.

We then assessed apoptosis in the three groups following HBO exposure using Annexin V-PE staining. At 0 h post-exposure, no significant differences were observed among the three groups (Figure 3d). However, at 6 h post-exposure, the HBO group exhibited a marked increase in apoptosis, with the percentage of annexin V-PE-positive cells rising from 21.8% in the NC group to 55.2% in the HBO group. In contrast, the HNO group showed no significant change (20.5%) compared to NC controls (Figure 3e). Collectively, these findings demonstrate that HBO exposure—but not HNO—induces mitochondrial depolarization, elevates mitochondrial ROS production, and triggers apoptosis in HT22 cells at 6 h post-exposure, consistent with the in vivo observations.

### 2.4. Mitophagy Is Progressively Induced but Delayed Following HBO Exposure, and Its Pre-Activation Alleviates Mitochondrial Damage and Apoptosis

**Figure 4:**
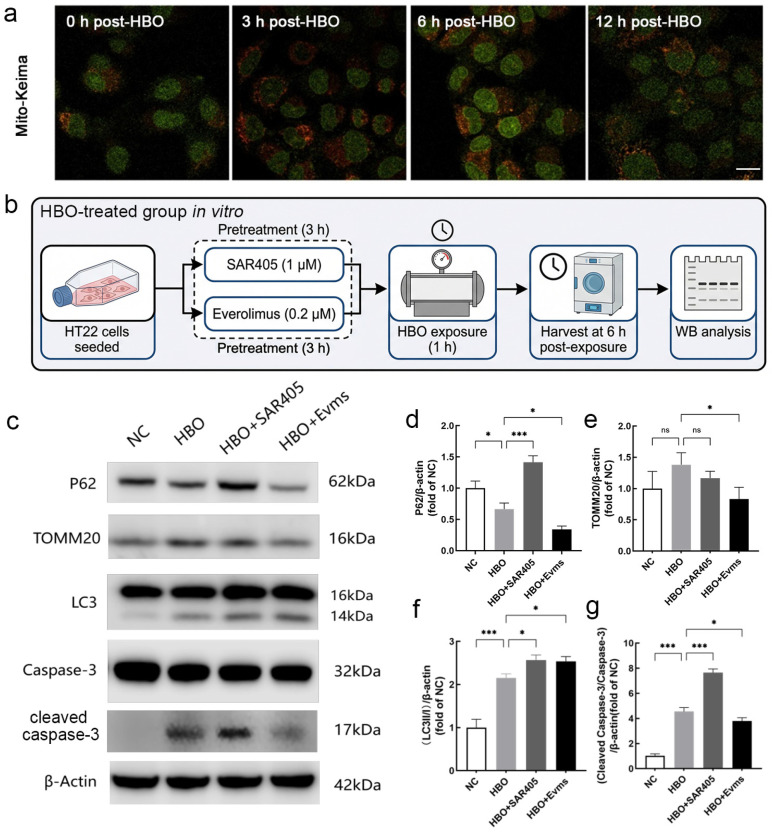
Mitophagy is progressively induced but delayed following HBO exposure, and its pre-activation alleviates mitochondrial damage and apoptosis: (**a**) Confocal microscopy images of Mito-Keima fluorescence in HeLa cells stably expressing parkin and Mito-Keima at 0 h, 3 h, 6 h, and 12 h after HBO exposure. Images show Mito-Keima fluorescence under 405 nm excitation (green) and 561 nm excitation (red). Scale bar, 20 μm. (**b**) Experimental timeline for the HBO-treated group in vitro. The NC group is not depicted in this timeline but was processed in parallel under identical sampling time points. (**c**–**g**) Western blot analysis of HT22 cells under the indicated conditions. Cells were pretreated with SAR405 (1 μM) or everolimus (0.2 μM) for 3 h, followed by HBO exposure for 1 h, and harvested at 6 h post-exposure. Representative immunoblots (**c**) and quantitative analysis of p62 (**d**), TOMM20 (**e**), LC3-II/LC3-I ratio (**f**), and cleaved caspase-3/caspase-3 ratio (**g**). Data are presented as mean ± SD from three independent experiments. * *p* < 0.05, *** *p* < 0.001 vs. indicated groups. NC, normobaric normoxia; HBO, hyperbaric oxygen; ns: not significant.

Given the observation that autophagosome-like membranes were recruited to damaged mitochondria following HBO exposure in vivo (Figure 1a), we next investigated whether this morphological event translates into functional mitophagy and whether it is sufficient to clear damaged mitochondria. To preliminarily explore the time course of mitophagy induction and dynamically monitor mitophagic flux, we utilized HeLa cells stably expressing parkin and the pH-sensitive mitophagy probe Mito-Keima [20,21]. Cells were exposed to HBO for 1 h, and Mito-Keima fluorescence was observed by confocal microscopy at various time points after decompression. As shown in Figure 4a, mitophagy was detectable at 3 h post-exposure and became markedly increased at 6 h, indicating that mitophagy is progressively induced following HBO exposure.

To further characterize the autophagic response in hippocampal neurons, HT22 cells were exposed to HBO for 1 h, and cell lysates were collected at 6 h post-exposure for Western blot analysis. Compared to the NC group, the HBO group exhibited a significant increase in the LC3-II/LC3-I ratio and a decrease in P62 expression, indicating activation of autophagic flux. However, TOMM20 showed no significant reduction in the HBO group (Figure 4c–f). These findings suggest that although autophagy is induced by 6 h post-exposure, the clearance of damaged mitochondria lags behind the injury process. The time required for mitophagic induction and completion allows damaged mitochondria to accumulate in the interim, and by the time autophagic flux is fully engaged, the window for effective clearance may have already passed.

We next examined how modulation of mitophagy affects HBO-induced apoptosis. HT22 cells were pretreated with SAR405, a Vps34 inhibitor that blocks autophagosome formation, or everolimus, an mTOR inhibitor and autophagy activator, for 3 h, followed by HBO exposure for 1 h. Protein expression was assessed at 6 h post-exposure by Western blotting (Figure 4b). Compared to the NC group, the HBO group showed decreased P62 expression, an increased LC3-II/LC3-I ratio, and an elevated cleaved caspase-3/caspase-3 ratio, with no significant change in TOMM20. Notably, SAR405 pretreatment further increased P62 expression and the cleaved caspase-3/caspase-3 ratio, while TOMM20 expression remained unchanged (Figure 4c–g). This indicates that SAR405 blocked autophagic flux and exacerbated apoptosis. The LC3-II/LC3-I ratio did not show inhibition when compared to the HBO group, which may be attributed to the combined effects of increased apoptosis and autophagic blockade. In contrast, everolimus pretreatment significantly decreased P62 and TOMM20 expression while further increasing the LC3-II/LC3-I ratio, indicating enhanced autophagic flux and successful clearance of damaged mitochondria, accompanied by a marked reduction in the cleaved caspase-3/caspase-3 ratio (Figure 4c–g). Of note, CCK-8 assay confirmed that treatment with SAR405 or everolimus alone at the indicated concentrations for 10 h had no cytotoxicity (Figure S1). Collectively, these results demonstrate that although HBO exposure triggers mitophagy, the endogenous response is initiated with a temporal delay, allowing damaged mitochondria to accumulate before clearance mechanisms become fully operational. Pre-activation of mitophagy with everolimus effectively eliminates damaged mitochondria in a timely manner and protects against apoptosis, whereas blockade of autophagic flux exacerbates cell death.

### 2.5. Pre-Activation of Mitophagy Alleviates HBO-Induced Mitochondrial Dysfunction, Apoptosis, and Prolongs Seizure Latency in Rats

**Figure 5:**
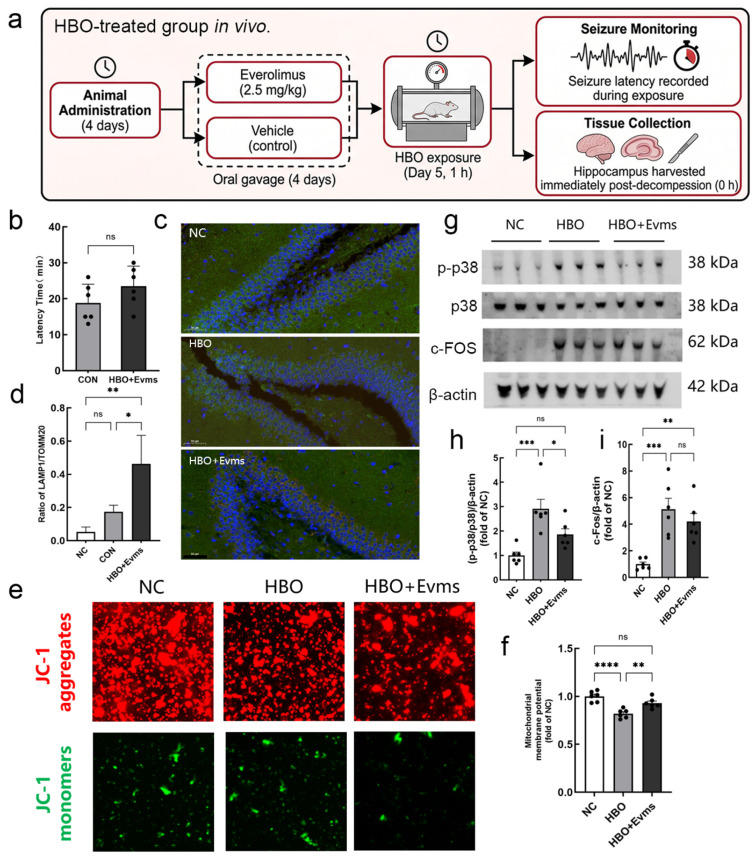
Pre-activation of mitophagy alleviates HBO-induced mitochondrial dysfunction and apoptosis and prolongs seizure latency in rats: (**a**) Experimental timeline for the HBO-treated group in vivo. The NC group is not depicted in this timeline but was processed in parallel under identical sampling time points. (**b**) Seizure latency recorded during HBO exposure. (**c**,**d**) Immunofluorescence staining of LAMP1 (red) and TOMM20 (green) in the rat hippocampus at 0 h after HBO exposure. Representative images (**c**) and quantitative analysis of colocalization (**d**). Scale bar: 50 μm. (**e**,**f**) Assessment of mitochondrial membrane potential using JC-1 staining. Representative fluorescence images (**e**) and quantitative analysis of the red/green fluorescence ratio (**f**). Scale bar: 400 μm. (**g**–**i**) Western blot analysis of p-p38, p38, c-Fos, and β-actin in the rat hippocampus. Representative immunoblots (**g**) and quantitative analysis of the p-p38/p38 ratio (**h**) and the c-Fos/β-actin ratio (**i**). Data are presented as mean ± SEM. * *p* < 0.05, ** *p* < 0.01, *** *p* < 0.001, **** *p* < 0.0001 vs. indicated groups, n = 6 per group. NC, normobaric normoxia; HBO, hyperbaric oxygen; EVMS, everolimus; ns: not significant.

We next investigated whether everolimus exerts similar protective effects in vivo. Rats were pretreated with everolimus (2.5 mg/kg) or vehicle by oral gavage once daily for 4 consecutive days, followed by HBO exposure on day 5. Seizure latency was recorded during exposure, and hippocampal tissues were collected immediately after decompression (0 h) for analysis (Figure 5a). Notably, everolimus pretreatment, to some extent, prolonged seizure latency compared to vehicle-treated controls (Figure 5b). Immunofluorescence staining for lysosome-associated membrane protein 1 (LAMP1), a lysosomal marker, and TOMM20 revealed that the HBO group exhibited limited colocalization at 0 h, indicating that endogenous mitophagic flux was not yet fully engaged; conversely, everolimus pretreatment markedly increased LAMP1/TOMM20 colocalization (Figure 5c,d), demonstrating that everolimus establishes a baseline of activated mitophagy, enabling immediate delivery of damaged mitochondria to lysosomes for degradation upon HBO exposure.

To assess whether everolimus preserves mitochondrial function and suppresses MAPK signaling, we measured ΔΨm using JC-1 staining and examined p-p38 and c-Fos expression by Western blotting. As shown in Figure 5e,f, the HBO-induced reduction in the red/green fluorescence ratio was significantly attenuated by everolimus pretreatment, indicating protection against mitochondrial depolarization. Additionally, HBO exposure significantly increased p-p38 and c-Fos levels, and both increases were significantly reduced by everolimus pretreatment (Figure 5g–i).

## 3. Discussion

In this study, we systematically investigated the role of mitophagy in CNS-OT. Our findings demonstrate that HBO exposure causes severe mitochondrial damage, disrupts mitochondrial membrane potential (ΔΨm), and activates pro-apoptotic MAPK signaling in hippocampal neurons. Although endogenous mitophagy is initiated following HBO exposure, it is markedly delayed, leading to the accumulation of dysfunctional mitochondria and subsequent neuronal injury. Importantly, pharmacological pre-activation of mitophagy with everolimus effectively eliminates damaged mitochondria, suppresses p-p38-mediated apoptosis, prolongs seizure latency, and protects against CNS-OT. Together, these results identify a critical time window during which mitochondrial quality control fails, and they establish mitophagy preconditioning as a promising preventive strategy.

Mitochondria are both primary targets and major sources of reactive oxygen species under hyperbaric hyperoxic conditions. Our transmission electron microscopy revealed that HBO, but not HNO, caused pronounced mitochondrial structural damage, including swelling, cristae disorganization, and matrix vacuolization, indicating that hyperoxia rather than hyperbaric pressure per se is the primary trigger of mitochondrial injury. These morphological changes correlated with a significant loss of ΔΨm, elevated mitochondrial and total ROS levels, and increased apoptosis at 6 h post-exposure. The selective enrichment of the MAPK, TNF, and NF-κB signaling pathways in our transcriptomic analysis further supports the notion that mitochondrial dysfunction is an upstream driver of the inflammatory and cell death programs observed in CNS-OT. The p38 MAPK pathway, in particular, is known to be activated by oxidative stress and can directly promote apoptosis by phosphorylating pro-apoptotic proteins. Our finding that p-p38 and c-Fos were upregulated in HBO-exposed animals and that these changes were attenuated by everolimus places mitochondrial quality control upstream of this pro-apoptotic signaling cascade.

Previous work has reported increased levels of mitophagy-related proteins (PINK1, LC3-II) in the hippocampus after HBO exposure [22], but whether these changes reflect functional mitophagic flux remained unclear. A central finding of this study is that while HBO exposure triggers mitophagic flux, the endogenous response is not timely enough to prevent neuronal injury because it lags behind the rapid onset of mitochondrial damage. Using the pH-sensitive Mito-Keima probe, we observed that mitophagic flux became detectable only at 3 h and markedly increased by 6 h after exposure. However, at the same 6 h time point, levels of the mitochondrial outer membrane protein TOMM20 remained unchanged, indicating that although autophagosome formation was enhanced, the clearance of damaged mitochondria had not yet been completed. This delay creates a window during which depolarized, ROS-generating mitochondria persist and can trigger apoptotic signaling. Furthermore, future work should systematically examine changes in other outer mitochondrial membrane proteins to better characterize the specificity and extent of mitophagic degradation. In addition, it will be important to explore HBO-specific mitophagy receptors and the distinctive regulatory patterns of mitophagy under hyperbaric hyperoxia.

Based on the observation that endogenous mitophagy is too slow to prevent injury, we tested whether pre-activating the mitophagy machinery could confer protection. Pretreatment with everolimus, an mTOR inhibitor and autophagy activator, to some extent prolonged seizure latency—a key behavioral manifestation of CNS-OT. Mechanistically, everolimus markedly enhanced colocalization of the lysosomal marker LAMP1 with TOMM20 immediately after HBO exposure, demonstrating that a baseline of activated mitophagy enables rapid delivery of damaged mitochondria to lysosomes. This timely clearance was reflected by a significant reduction in TOMM20 protein levels in everolimus-treated cells and animals, indicating successful elimination of dysfunctional mitochondria. Importantly, everolimus pretreatment also prevented the HBO-induced loss of ΔΨm and suppressed the activation of p-p38 MAPK and c-Fos, linking efficient mitophagy to the inhibition of pro-apoptotic signaling. Conversely, inhibition of autophagy with SAR405 blocked autophagic flux, increased TOMM20 accumulation, and exacerbated apoptosis, further confirming that adequate mitophagic clearance is essential for cell survival under hyperbaric hyperoxic stress.

CNS-OT remains a major limitation to the clinical use of HBO and poses a significant risk to divers, hyperbaric personnel, and patients undergoing HBO therapy. Traditional countermeasures primarily rely on removing the individual from the hyperbaric environment and administering symptomatic anticonvulsant treatment. However, in special operational scenarios where immediate removal from the stressful environment is not feasible, there is an urgent need for fundamental strategies that can delay or prevent the onset of toxicity. Everolimus and other mTOR inhibitors are already clinically approved for other indications, raising the possibility of repurposing them for prophylactic use. Nevertheless, several important questions remain before such an approach can be considered. Although everolimus was effective in our acute exposure model, the potential side effects of even short-term mTOR inhibition must be carefully weighed against the benefits of seizure prevention. Future studies should explore alternative mitophagy activators with improved safety profiles or more selective actions. Addressing these questions will not only deepen our mechanistic understanding but also pave the way for rational design of preventive interventions against hyperbaric oxygen neurotoxicity.

## 4. Materials and Methods

### 4.1. Animal Housing and Grouping

Male Sprague Dawley rats weighing 200–250 g (approximately 8 weeks of age) were purchased from Bikai Keyi Biotechnology Co., Ltd. (Shanghai, China). All animal procedures were approved by the Institutional Animal Care and Use Committee and were conducted in accordance with the National Institutes of Health Guide for the Care and Use of Laboratory Animals. Animals were housed under a 12 h light/dark cycle with free access to food and water.

To distinguish the effects of hyperbaric pressure from those of hyperoxia, a total of 18 rats were randomly assigned into three groups (n = 6 per group): normobaric normoxia (NC) group, hyperbaric normoxia (HNO) group, and hyperbaric oxygen (HBO) group. Hippocampal tissues from the NC and HBO groups were collected for RNA sequencing analysis, as the HNO group did not exhibit mitochondrial damage. To evaluate the protective effect of everolimus pretreatment, another 18 rats were randomly divided into three groups (n = 6 per group): NC group, vehicle-treated HBO group, and everolimus-treated HBO group. Seizure latency was recorded in the two HBO-exposed groups. For everolimus pretreatment, rats in the everolimus-treated HBO group received intragastric administration of 2.5 mg/kg everolimus (Macklin (Shanghai, China), 159351-69-6) suspended in 0.5% sodium carboxymethyl cellulose (CMC-Na) once daily for four consecutive days. Rats in the vehicle-treated HBO group received an equal volume of 0.5% CMC-Na solution following the same schedule. The dose of 2.5 mg/kg was selected based on previous studies demonstrating effective autophagy activation and neuroprotection in vivo [23].

### 4.2. In Vivo Hyperbaric Exposure

Based on preliminary experiments in our laboratory, rats were placed in the animal hyperbaric chamber (customized by Hongyuan Oxygen Industrial (Yantai, China)) and exposed to 100% oxygen at 0.6 MPa (6 ATA) for 30 min (HBO). During the exposure, animals were continuously monitored for convulsions, consciousness, and respiration and were removed from the chamber by decompression at 0.1 MPa/min either immediately upon prolonged loss of consciousness or after completion of the fixed 30 min exposure, whichever occurred first. For hyperbaric normoxia (HNO) controls, rats were exposed to a nitrogen–oxygen mixture at 0.6 MPa with a normobaric oxygen partial pressure. The normobaric normoxia (NC) group was kept at room air under normal atmospheric pressure.

### 4.3. Transmission Electron Microscopy

The procedure was performed as previously described [24]. Hippocampal tissues were fixed in 2.5% glutaraldehyde, post-fixed in 1% osmium tetroxide, dehydrated through a graded ethanol series, and embedded in epoxy resin. Ultrathin sections (60 nm) were stained with uranyl acetate and lead citrate, then examined under a transmission electron microscope.

### 4.4. RNA Sequencing and Transcriptomic Analysis

Total RNA was extracted from hippocampal tissues of the NC and HBO groups using TRIzol reagent (Invitrogen (Carlsbad, CA, USA), 15596026). RNA purity and concentration were assessed using a NanoDrop spectrophotometer (Wilmington, DE, USA). Libraries were constructed and sequenced on an Illumina NovaSeq 6000 platform (San Diego, CA, USA). Differential expression analysis was performed using DESeq2, with genes showing adjusted *p*-value < 0.05 considered significantly differentially expressed. KEGG pathway enrichment and Gene Ontology (GO) analysis were conducted using clusterProfiler (v4.14.6).

### 4.5. Cell Culture

HT22 mouse hippocampal neurons were cultured in a specialized medium optimized for this cell line (ServiceBio (Wuhan, China), GZ20011) consisting of DMEM (ServiceBio, G4511) supplemented with 10% fetal bovine serum (ServiceBio, G8002) and 1% penicillin–streptomycin (ServiceBio, G4003). HeLa cells stably expressing parkin and Mito-Keima were generated by retroviral transduction as previously described [20,21] and were a generous gift from Prof. Jiahong Lu at the University of Macau. Cells were maintained at 37 °C in a humidified atmosphere containing 5% CO_2_.

### 4.6. In Vitro Hyperbaric Exposure

HT22 cells or HeLa cells were placed in the cell hyperbaric chamber (customized by Hongyuan Oxygen Industrial) and exposed to 100% oxygen at 0.6 MPa for 1 h. After decompression, cells were collected at various time points (0 h, 3 h, or 6 h) for further analysis. HNO and NC groups were treated similarly as described above.

### 4.7. Measurement of ROS

Total ROS levels were assessed using DCFH-DA (Beyotime (Shanghai, China), S0034S). According to the manufacturer’s instructions, HT22 cells were incubated with 10 μM DCFH-DA at 37 °C for 30 min, and fluorescence was analyzed by flow cytometry. Mitochondrial ROS were measured using MitoSOX Red (Beyotime, S0061S). Cells were incubated with 5 μM MitoSOX Red at 37 °C for 10 min following the manufacturer’s protocol, and fluorescence was analyzed by flow cytometry.

### 4.8. Annexin V-PE Apoptosis Detection

According to the manufacturer’s instructions, HT22 cells were harvested at 0 h or 6 h after HBO exposure, washed with PBS, and stained using the Annexin V-PE Apoptosis Detection Kit (Beyotime, C1065S). Samples were incubated at room temperature for 15 min in the dark, and fluorescence was analyzed by flow cytometry.

### 4.9. Measurement of Mitochondrial Membrane Potential (*Δ*Ψm)

ΔΨm was assessed using JC-1 staining (Beyotime, C2003S) for isolated mitochondria and TMRE staining (Macklin (Shanghai, China), 115532-52-0) for cultured cells. For JC-1, mitochondria were isolated from hippocampal tissues and stained according to the manufacturer’s instructions. Fluorescence was observed under a fluorescence microscope, and the red/green fluorescence ratio was calculated to represent ΔΨm. For TMRE, HT22 cells were stained following previously described methods [25], and fluorescence intensity was analyzed by flow cytometry.

### 4.10. Western Blotting

Western blotting was performed according to the method described previously [26]. In brief, hippocampal tissues or HT22 cells were lysed in RIPA buffer containing protease and phosphatase inhibitors. Protein concentrations were determined using the BCA assay (Thermo Scientific (Waltham, MA, USA), 23225). Equal amounts of protein were separated by SDS-PAGE and transferred to PVDF membranes. Membranes were blocked with 5% non-fat milk and incubated overnight at 4 °C with primary antibodies against LC3B (AiFang biological, SHDW251215), p62 (Abclone (Wuhan, China), A19700), TOMM20 (Abclone, A19403), caspase-3 (Abclone, A19654), p-p38 (Abclone, AP0526), p38 (Abclone, A4771), c-Fos (Abclone, A24620), and β-actin (Abclone, AC026). After incubation with HRP-conjugated secondary antibodies (CST (Danvers, MA, USA), 7074P2), signals were visualized using enhanced chemiluminescence (MCE (South Brunswick Township, NJ, USA), HY-K1005) and quantified using ImageJ software (v1.54f).

### 4.11. Immunofluorescence and Confocal Microscopy

Immunofluorescence was performed according to the method described previously [26]. For colocalization analysis, hippocampal sections were fixed with 4% paraformaldehyde, permeabilized with 0.1% Triton X-100, and blocked with 10% normal goat serum. Samples were incubated with primary antibodies against LAMP1 (ServiceBio (Wuhan, China), GB112949) and TOMM20 (Selleckchem (Houston, TX, USA), F0513) overnight at 4 °C, followed by incubation with Alexa Fluor-conjugated secondary antibodies. Nuclei were counterstained with DAPI. Images were acquired using a confocal laser scanning microscope (Zeiss (Oberkochen, Germany), LSM900), and colocalization was quantified using ImageJ software.

### 4.12. Statistical Analysis

All data are presented as mean ± standard error of the mean (SEM) from at least three independent experiments. Statistical analyses were performed using GraphPad Prism 8.0. Comparisons between two groups were conducted using a two-tailed Student’s *t*-test. Comparisons among multiple groups were performed using one-way ANOVA followed by Tukey’s post hoc test. A *p*-value < 0.05 was considered statistically significant.

## 5. Conclusions

In conclusion, this study demonstrates that HBO exposure induces severe mitochondrial damage and activates pro-apoptotic MAPK signaling in the hippocampus. Although endogenous mitophagy is initiated, its delayed occurrence allows dysfunctional mitochondria to accumulate, leading to neuronal injury. Pre-activation of mitophagy with everolimus primes the system for timely clearance of damaged mitochondria, preserves mitochondrial membrane potential, suppresses p-p38-mediated apoptosis, and protects against CNS-OT. These findings establish mitophagy pre-activation as a novel and promising strategy to prevent central nervous system oxygen toxicity, suggesting its potential application in hyperbaric clinical or operational settings.

## Data Availability

The original contributions presented in this study are included in the article. Further inquiries can be directed to the corresponding author.

## Supplementary Materials

The following supporting information can be downloaded at: https://www.mdpi.com/article/10.3390/ijms27114982/s1.

## Abbreviations

The following abbreviations are used in this manuscript:

ΔΨm	Mitochondrial membrane potential
CNS-OT	Central nervous system oxygen toxicity
FoxO	Forkhead box O
FUNDC1	FUN14 domain containing 1
GO	Gene ontology
HBO	Hyperbaric oxygen
HNO	Hyperbaric normoxia
KEGG	Kyoto Encyclopedia of Genes and Genomes
LAMP1	Lysosome-associated membrane protein 1
LC3	Microtubule-associated protein 1 light chain 3
MAPK	Mitogen-activated protein kinase
MMP	Mitochondrial membrane potential
NC	Normobaric normoxia
NF-κB	Nuclear factor kappa B
PINK1	PTEN-induced putative kinase 1
ROS	Reactive oxygen species
TMRE	Tetramethylrhodamine ethyl ester
TNF	Tumor necrosis factor
TOMM20	Translocase of outer mitochondrial membrane 20
