# Supplementary material for: Pre-Activation of Mitophagy Protects Against Hyperbaric Oxygen-Induced Central Nervous System Oxygen Toxicity
Source: Int J Mol Sci. 2026 May 30;27(11):4982. doi: 10.3390/ijms27114982 (PMC13256620; doi:10.3390/ijms27114982)
Supplement: Supplementary file 1 [file ijms-27-04982-s001.zip › ijms-4262547-supplementary.pdf]

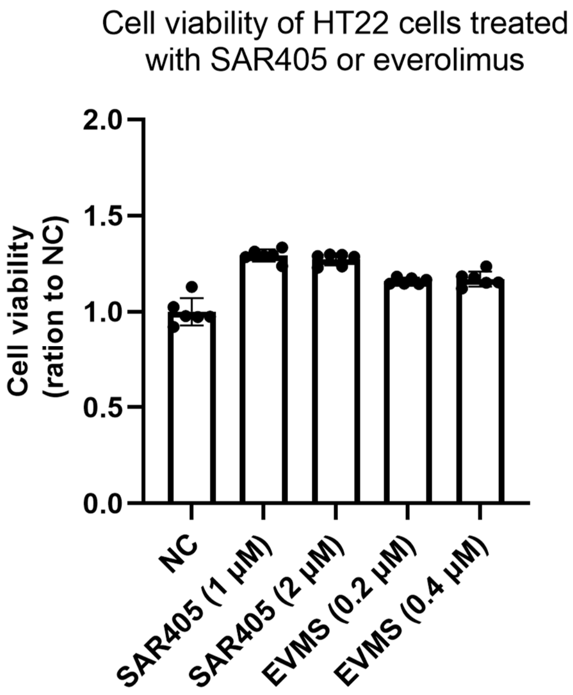

Figure S1. Cell viability of HT22 cells treated with SAR405 or everolimus. HT22 cells were treated with the indicated concentrations of SAR405 (1, 2 μM) or everolimus (0.2, 0.4 μM) for 10 h. Viability was assessed by the CCK8 assay. No reduction in viability was observed in any treatment group relative to the NC group (n = 6).
